# The recomBead Borrelia antibody index, CXCL13 and total IgM index for laboratory diagnosis of Lyme neuroborreliosis in children

**DOI:** 10.1007/s10096-017-3049-x

**Published:** 2017-07-20

**Authors:** B. H. Skogman, M. Lager, A. J. Henningsson, I. Tjernberg

**Affiliations:** 1 Center for Clinical Research (CKF) Dalarna, Uppsala University, Nissers väg 3, 791 82 Falun, Sweden; 2 Laboratory Medicine, Region Jönköping County, Jönköping, Sweden; 3 Clinical Microbiology, Region Jönköping County, Jönköping, Sweden; 4 Department of Clinical Chemistry and Transfusion Medicine, Kalmar County Council, Kalmar, Sweden; 5 Department of Clinical and Experimental Medicine, Linköping University, Linköping, Sweden

## Abstract

For laboratory diagnostics of Lyme neuroborreliosis (LNB), the recomBead Borrelia antibody index (AI) assay has shown promising results in a mixed age population, but has not previously been evaluated with specific focus on paediatric patients. The aim of the study was to evaluate the recomBead Borrelia AI assay in cerebrospinal fluid (CSF) for the laboratory diagnosis of LNB in children. We also wanted to explore whether early markers, such as CXCL13 in CSF and/or total IgM index could be useful as complementary diagnostic tools. Children being evaluated for LNB in a Swedish Lyme endemic area were included in the study (*n* = 146). Serum and CSF were collected on admission. Patients with other specific diagnoses were controls (*n* = 15). The recomBead Borrelia AI assay and the recomBead CXCL13 assay (Mikrogen) were applied together with total IgM index. The overall sensitivity for recomBead Borrelia AI (IgM and IgG together) was 74% and the specificity was 97%. However, the highest sensitivity (91%) at an acceptable level of specificity (90%) was obtained by recomBead Borrelia AI together with CXCL13 and total IgM index, showing a positive predictive value of 84% and a negative predictive value of 95%. Thus, the recomBead Borrelia AI assay performs with moderate sensitivity and high specificity in paediatric LNB patients. The major advantage seems to be increased sensitivity in the possible LNB group compared to the IDEIA assay. The diagnostic sensitivity may be further increased by using a combination of early markers, such as CXCL13 in CSF and total IgM index.

## Introduction

Laboratory diagnosis of Lyme neuroborreliosis (LNB) has been hampered over the years with observed heterogeneity and unsatisfactory diagnostic accuracy [1]. The first generation of anti-*Borrelia* antibody tests, based on whole-cell sonicates, showed low specificity [2]. The second generation of antibody tests in serum/cerebrospinal fluid (CSF), based on purified native antigens, such as the flagella antigen, showed a higher specificity but could not distinguish between present and previous LNB due to prolonged antibody detection in CSF [3, 4]. Furthermore, second-generation antibody tests may show an unsatisfactory sensitivity in patients with early LNB but are still considered the main diagnostic tool, together with CSF cell count and clinical assessment [5]. Paediatric patients often present as early LNB with short duration of symptoms, and antibody production in CSF may not always be present at the time of lumbar puncture [6–8]. The third generation of antibody tests, based on recombinant *Borrelia* antigens and/or purified peptides, has demonstrated a slightly higher sensitivity with a maintained specificity in laboratory diagnosis of LNB [1]. The recomBead Borrelia antibody index (AI) assay, a Luminex-based multiplex bead assay with several recombinant antigens included, has shown promising results in a mixed age population [9], but has not previously been evaluated with specific focus on paediatric patients.

As an early diagnostic marker, the B cell-attracting chemokine CXCL13 has been proven to be reliably elevated in the CSF of patients with early LNB [10–17]. The chemokine CXCL13 is, therefore, suitable for laboratory diagnostics of LNB in paediatric patients [13, 18]. Furthermore, it has been suggested that the total IgM index (serum/CSF) can be useful as an unspecific inflammatory marker in laboratory diagnostics of LNB [15, 19], but the total IgM index has not previously been evaluated in paediatric patients.

The aim of the study was to evaluate the recomBead Borrelia AI assay in laboratory diagnostics of LNB in children. We also wanted to explore whether early markers, such as CXCL13 and/or total IgM index, could be useful as complementary diagnostic tools for LNB in paediatric patients.

## Materials and methods

### Patients and controls

Children being evaluated for LNB at seven paediatric departments in a Lyme endemic area in Central and Southeast Sweden during the years 2010–2013 accepted to participate and were consecutively included in the study. On admission, the child and parents/guardians completed a standardised questionnaire with questions concerning current symptoms, observed tick bites, previous antibiotic treatment of Lyme borreliosis (LB) and the basic health of the child. Serum and CSF samples were taken on admission, i.e. before the start of antibiotic treatment. Of 153 patients included in the study, seven children were excluded because of missing data. These children (*n* = 7) did not differ in median age or gender from patients included in the study (*n* = 146).

Patients with other specific diagnoses during the study period, who underwent a lumbar puncture, were asked, together with parents/guardians, to participate in the study and were enrolled as control patients (*n* = 15). The child and parents/guardians completed a standardised questionnaire for control patients, and CSF and blood samples were taken for laboratory analyses.

### Classification of LNB patients and controls

LNB patients were classified as definite LNB or possible LNB in accordance with European guidelines (Table 1) [20]. All LNB patients had neurological symptoms indicative of LNB without other obvious reasons, pleocytosis in CSF with mononuclear dominance (data not shown) and unambiguous response to antibiotic treatment. Intrathecal anti-*Borrelia* antibody production (IgG and IgM) was analysed in all patients as part of clinical routine, with a flagella antigen-based enzyme-linked immunosorbent assay (ELISA) according to the manufacturer’s instructions (IDEIA Lyme Neuroborreliosis Kit, Oxoid, Hampshire, UK) [4]. Pleocytosis was defined as total cell count >5 × 10^6^/L in CSF [21]. Serum and CSF samples were collected during 2010–2013 and stored at −70 °C without being thawed, until analysis for recomBead AI, CXCL13 and total IgM index (see below) during 2015.

**Table 1:** Classification of LNB patients and controls

Diagnosis	Criteria
LNB patients (*n* = 58)	Definite LNB (*n* = 37)^a^ 1. Neurological symptoms indicative of LNB without other obvious reasons 2. Pleocytosis in CSF^b^ 3. Intrathecal anti-*Borrelia* antibodies (IgG and/or IgM)^c^
	Possible LNB (*n* = 21)^a^ Criteria 1 and 2 are fulfilled
Controls (*n* = 103)	Non-LNB (*n* = 88) Patients not meeting the criteria for definite LNB or possible LNB
	Other diagnosis (*n* = 15) Patients with other specific diagnosis

LNB = Lyme neuroborreliosis; CSF = cerebrospinal fluid; Ig = immunoglobulin; *n* = number

^a^LNB patients classified in accordance with European guidelines [20]

^b^Total cell count >5 × 10^6^/L in CSF

^c^Detected by the IDEIA Lyme Neuroborreliosis neuroborreliosis assay [4]

Patients who did not meet the criteria for either definite LNB or possible LNB were classified as non-LNB and patients with other specific diagnoses were classified as other diagnosis (Table 1). The non-LNB and other diagnosis groups were all negative for anti-*Borrelia* antibodies in CSF (IDEIA, Oxoid) and were considered as controls.

### Clinical characteristics of LNB patients and controls

Clinical characteristics and laboratory data from *all* patients (*n* = 146) being evaluated for LNB (definite LNB, possible LNB and non-LNB) are shown in Table 2. Fatigue, headache and acute peripheral facial nerve palsy were major clinical manifestations and known tick bite was reported in 52%. Antibiotics for LNB patients were given in doses according to the Swedish Medical Products Agency (national guidelines available at https://lakemedelsverket.se/upload/halso-och-sjukvard/behandlingsrekommendationer/Borrelia-rek_webb_bokm%c3%a4rken.pdf); i.e. ceftriaxone i.v. 50–100 mg/kg/dos once daily in 10–14 days for children <8 years of age and doxycycline p.o. 4 mg/kg once daily in 10–14 days for children 8 years of age or older. Some of the patients also presented with a cutaneous manifestation, such as erythema migrans (EM) or lymphocytoma (*n* = 23) (Table 2).

**Table 2:** Characteristics of *all* children being evaluated for LNB

On admission	Patients (*n* = 146)
Age, median, years (range)	10 (2–18)
Gender	
Female, *n* (%)	83 (56)
Male, *n* (%)	64 (44)
Known tick bite, *n* (%)	77 (52)
Major clinical features	
Fatigue, *n* (%)	109 (74)
Headache, *n* (%)	106 (72)
Facial nerve palsy, *n* (%)	70 (48)
Fever, *n* (%)	66 (45)
Loss of appetite, *n* (%)	63 (43)
Neck pain, *n* (%)	56 (38)
Vertigo, *n* (%)	54 (37)
Nausea, *n* (%)	54 (37)
Neck stiffness, *n* (%)	31 (21)
Radiating pain, *n* (%)	30 (20)
Erythema migrans (EM) and/or lymphocytoma, *n* (%)	37 (25)
Laboratory findings	
Pleocytosis in CSF, *n* (%)^a^	58 (39)
Total cell count × 106/L cells, median (range)	132 (8–890)
Anti- *﻿Borrelia* ﻿antibodies in CSF, *n* (%)^b^	37 (25)
IgM, n (%)	5 (13)
IgG, n (%)	8 (22)
IgM + IgG, n (%)	24 (65)
Anti-*Borrelia* antibodies in serum, *n* (%)	55 (37)
IgM, n (%)	14 (9)
IgG, n (%)	15 (10)
IgM + IgG, n (%)	26 (16)
Antibiotic treatment, n (%)	76 (52)
Diagnosis	
Definite LNB, *n* (%)^c^	37 (25)
Possible LNB, *n* (%)^c^	21 (14)
Non-LNB, *n* (%)	88 (61)

LNB = Lyme neuroborreliosis; CSF = cerebrospinal fluid; Ig = immunoglobulin; *n* (%) = number (percent). Patients could have one or more symptoms

^a^Total cell count >5 × 10^6^/L cells in CSF

^b^Detected by the IDEIA Lyme neuroborreliosis assay [4]

^c^LNB patients classified in accordance with European guidelines [20]

Children in the non-LNB group (*n* = 88) were patients presenting with acute facial nerve palsy (*n* = 30) or headache and unspecific symptoms (such as fatigue or problems with food intake or sleep) (*n* = 58). Non-LNB patients had no pleocytosis in CSF and no intrathecal anti-*Borrelia* antibodies, i.e. did not meet the criteria for definite LNB or possible LNB (Table 1) and are considered as controls. A few of the non-LNB patients received antibiotic treatment prescribed by their paediatrician due to the occurrence of EM and/or anti-*Borrelia* antibodies in serum (*n* = 14) or on vague/incorrect indication (*n* = 4).

Another control group was included in the study. Those were patients who received other specific diagnoses after lumbar puncture and further investigation was performed (*n* = 15) (Table 1). This control group consisted of patients who were diagnosed as clinical viral meningitis (*n* = 5) with high fever, meningeal signs and pleocytosis (two of them had enterovirus verified by PCR in CSF), post-infectious encephalitis (*n* = 1), idiopathic intracranial hypertension with headache (*n* = 4), stroke with hemiplegia (*n* = 1) and recurrent one-sided migraine headache (*n* = 4).

### The recomBead Borrelia AI assay

The recomBead Borrelia IgG/IgM assay (Mikrogen Diagnostik GmbH, Neuried, Germany), a multiplex bead array using Luminex xMAP technology and, in this study, a BioPlex 200 system (BioRad Laboratories, Inc., Hercules, CA, USA) was used together with the software Xponent, version 33.1.871.0 (Luminex Corporation, Austin, TX, USA). The test includes 13 recombinant *Borrelia* antigens attached to polystyrene beads [9]. Albumin, total IgM and total IgG in serum and CSF for the calculation of recomBead Borrelia AI were analysed by nephelometry with the Immage 800 instrument (Beckman Coulter, Fullerton, CA, USA) [9]. The recomBead Borrelia AI was calculated for each antigen separately according to Reiber and Peter (slightly modified) [22] by the Excel program available from Mikrogen Diagnostik. Cut-off levels and the overall assessments of the test results were used as recommended by the manufacturer and equivocal test results were regarded as positive in the subsequent calculations.

### The recomBead CXCL13 assay in CSF

The same Luminex-based platform as described above was used for the recomBead CXCL13 assay (Mikrogen Diagnostik) and applied according to the manufacturer’s instructions for evaluation of CXCL13 in CSF. The measuring range for CXCL13 was 9–1000 pg/mL, and sample results above 1000 pg/mL were not diluted and reanalysed, and were thus regarded as >1000 pg/mL. Equivocal test results, as defined by the manufacturer (190–300 pg/mL) were considered as positive. The cut-off was modified to 160 pg/mL based on a separate receiver operating characteristic (ROC) curve calculation on patients with definite LNB (*n* = 37) and non-LNB (*n* = 88) from this present material (data not shown).

### Calculation of total IgM index

The total IgM index was calculated as:

$$ \frac{\mathrm{CSF}-\mathrm{IgM}\left(\mathrm{mg}/\mathrm{L}\right)/\mathrm{S}-\mathrm{IgM}\left(\mathrm{g}/\mathrm{L}\right)}{\mathrm{CSF}-\mathrm{Albumin}\left(\mathrm{mg}/\mathrm{L}\right)/\mathrm{S}-\mathrm{Albumin}\ \left(\mathrm{g}/\mathrm{L}\right)} $$

The total IgM index was used as part of the recomBead Borrelia AI assay but also as a single IgM index calculation (as described above). The cut-off for the single IgM index was set at 0.148 mg/L, based on a separate ROC curve calculation on patients with definite LNB (*n* = 37) and non-LNB (*n* = 88) from this present material (data not shown).

### Statistics

SPSS software, version 21 (SPSS Inc., Chicago, IL, USA) was used for statistical calculations. A *p*-value <0.05 was considered significant. Mann–Whitney’s *U*-test was used for comparison of the median age between included/excluded patients. Fisher’s exact test was used for comparison of sex between included/excluded patients. For calculation of sensitivity of the diagnostic tests, patients classified as definite LNB and possible LNB (Table 1) were used as LNB patients, since they represent well-characterised clinical paediatric LNB cases with no other obvious diagnosis who responded unambiguously well to treatment. For calculation of specificity, controls classified as non-LNB and other diagnosis (Table 1) were used, since they represent relevant paediatric control patients without LNB and were all negative to anti-*Borrelia* antibodies in CSF (IDEIA, Oxoid). ROC curve analyses were made using MedCalc version 16.1 (MedCalc Software, Ostend, Belgium) and the area under the curve (AUC) was used for the calculation of diagnostic performance.

## Results

### The recomBead Borrelia AI assay

Among children with definite LNB, 33 out of 37 patients (89%) were positive with the recomBead Borrelia AI assay, whereas in the possible LNB group, 10 out of 21 patients (48%) were AI-positive (Table 3). In the non-LNB group, 89 out of 92 children (97%) were negative with the recomBead Borrelia AI assay and in the other diagnosis group, all children (100%) were AI-negative (Table 3).

**Table 3:** The recomBead Borrelia AI assay in the different patient groups

	LNB patients (*n* = 58)		Controls (*n* = 103)		Diagnostic performance
	Definite LNB (it.ab.+/pleo+) *n* = 37	Possible LNB (it.ab.−/pleo+) *﻿﻿n* = 21	Non-LNB (it.ab.−/pleo−) *n* = 88	Other diagnosis (it.ab.−/pleo−) *n* = 15	
recomBead AI:					
Positive AI, *n* (%)	33 (89)	10 (48)	3 (3)	0 (0)	Sensitivity: 74%
Negative AI, *n* (%)	4 (11)	11 (52)	85 (97)	15 (100)	Specificity: 97%

LNB = Lyme neuroborreliosis; AI = antibody index, i.e. intrathecally produced IgG and/or IgM anti-*Borrelia* antibodies detected by the recomBead assay; it.ab = intrathecally produced IgG and/or IgM anti-*Borrelia* antibodies detected by the IDEIA Lyme neuroborreliosis assay; pleo = pleocytosis in CSF (total cell count >5 × 10^6^/L); *n* (%) = number (percent)

The overall sensitivity for LNB patients (*n* = 58) was 74% for the recomBead Borrelia AI assay and the specificity for controls (*n* = 103) was 97% (Table 3).

### The recomBead Borrelia antibody assay in serum

Thirty-eight out of 58 (66%) LNB patients were positive in serum (IgG and/or IgM), which gives the recomBead Borrelia antibody assay in serum an overall sensitivity of 66% (data not shown). Ninety-two out of 103 (89%) controls were negative in serum (IgG and/or IgM), which gives a specificity of 89% (data not shown).

### The recomBead CXCL13 assay in CSF

In the definite LNB group, 36 out of 37 children (97%) were positive for CXCL13 in CSF (median 923 pg/mL) and in the possible LNB group, 15 out of 21 children (71%) were positive for CXCL13 (median 489 pg/mL) (Table 4). In the non-LNB group, 83 out of 88 (94%) patients were negative for CXCL13 in CSF (median 35 pg/mL) and among patients with other diagnosis, 13 out of 15 (87%) were negative for CXCL13 (median 47 pg/mL) (Table 4). The overall sensitivity for LNB patients (*n* = 58) was 88% for the recomBead CXCL13 assay in CSF and the specificity for controls (*n* = 103) was 93% (Table 4).

**Table 4:** The CXCL13 and total IgM index in different patient groups

	LNB patients (*n* = 58)		Controls (*n* = 103)		Diagnostic performance
	Definite LNB (it.ab.+/pleo+) *n* = 37	Possible LNB (it.ab.−/pleo+) *n* = 21	Non-LNB (it.ab.−/pleo−) *n* = 88	Other diagnosis (it.ab.−/pleo−) *n* = 15	
CXCL13:					
Positive test, *n* (%)	36 (97)	15 (71)	5 (6)	2 (13)	Sensitivity: 88%
Negative test, *n* (%)	1 (3)	6 (29)	83 (94)	13 (87)	Specificity: 93%
Total IgM^a^:					
Positive test, *n* (%)	37 (100)	12 (57)	0 (0)	2 (13)	Sensitivity: 84%
Negative test, *n* (%)	0 (0)	9 (43)	88 (100)	13 (87)	Specificity: 98%

LNB = Lyme neuroborreliosis, it.ab = intrathecally produced IgG and/or IgM anti-*Borrelia* antibodies detected by the IDEIA Lyme neuroborreliosis assay; pleo = pleocytosis in CSF (total cell count >5 × 10^6^/L); *n* (%) = number (percent)

^a^Index calculated from total IgM in serum and CSF

### The total IgM index in CSF/serum

The results for the total IgM index alone among different patient groups are shown in Table 4. The overall sensitivity for LNB patients (*n* = 58) was 84% and the specificity for controls (*n* = 103) was 98%.

### The recomBead Borrelia AI assay in combination with CXCL13 and total IgM index

As both recomBead CXCL13 in CSF and total IgM index are apparently sensitive markers for LNB (Table 4), they were combined with results from the recomBead Borrelia AI assay. For a positive combined test result, patients needed to have positive recomBead Borrelia AI and/or positive CXCL13 in CSF and/or positive total IgM index. For a negative combined test result, patients needed to be negative for all three tests. The results are shown in Table 5.

**Table 5:** The combination of recomBead Borrelia AI, CXCL13 and total IgM index in different patient groups

	LNB patients (*n* = 58)		Controls (*n* = 103)		Diagnostic performance
	Definite LNB (it.ab.+/pleo+) *n* = 37	Possible LNB (it.ab.−/ pleo+) *n* = 21	Non-LNB (it.ab.−/pleo−) *n* = 88	Other diagnosis (it.ab.−/pleo−) *n* = 15	
recomBead AI and/or CXCL13 and/or total IgM^a^:					
Positive test, *n* (%)	37 (100)	16 (76)	7 (8)	3 (20)	Sensitivity: 91%
Negative test, *n* (%)	0 (0)	5 (24)	81 (92)	12 (80)	Specificity: 90%

LNB = Lyme neuroborreliosis; AI = antibody index, i.e. intrathecally produced IgG and/or IgM anti-*Borrelia* antibodies detected by the recomBead assay; pleo = pleocytosis in CSF (total cell count >5 × 10^6^/L); it.ab = intrathecally produced IgG and/or IgM anti-*Borrelia* antibodies detected by the IDEIA Lyme neuroborreliosis assay; *n* (%) = number (percent)

^a^Index calculated from total IgM in serum and CSF

In definite LNB, 37 out of 37 children (100%) were positive with the combination of tests and in the possible LNB group, 16 out of 21 children (76%) were positive (Table 5). In non-LNB, 85 out of 92 (92%) were negative with the combination of tests and among patients with other diagnosis, 12 out of 15 (80%) were negative (Table 5). The diagnostic performance for this combination of tests showed an overall sensitivity for LNB patients (*n* = 58) of 91% and an overall specificity for controls (*n* = 103) of 90% (Table 5).

**Fig. 1:**
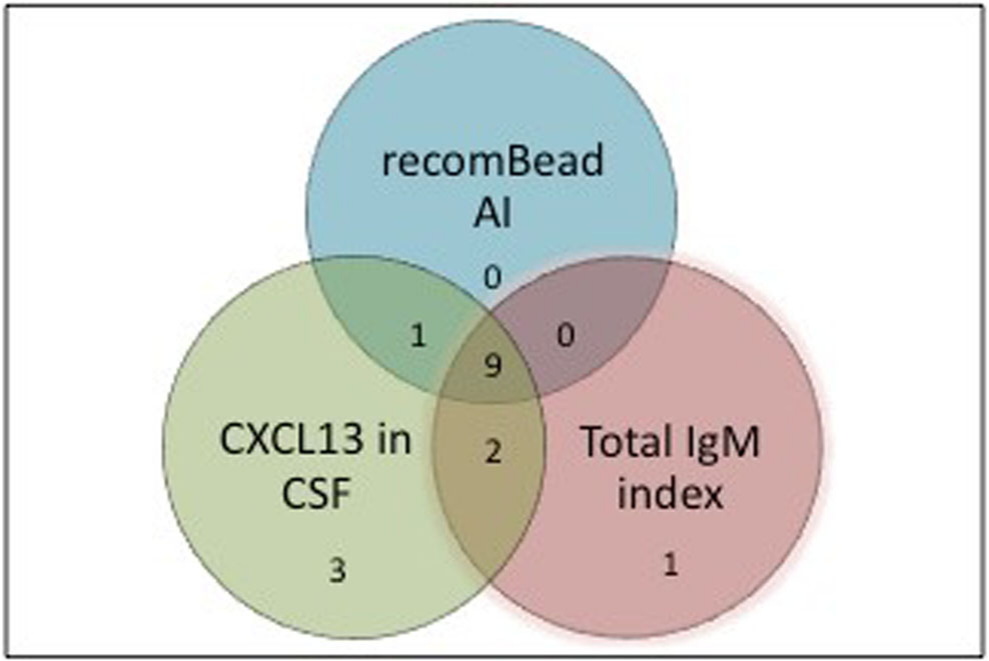
Concordance between positive test results for recomBead Borrelia AI, CXCL13 and total IgM index in possible LNB patients (16 out of 21)

In Fig. 1, the possible LNB group with pleocytosis is further visualised for the combination of different positive test results (16 out of 21). There was a strong concordance between recomBead Borrelia AI, CXCL13 and total IgM index, and only a few patients were positive for CXCL13 (*n* = 3) or total IgM index (*n* = 1) alone (Fig. 1). Serum antibodies for the recomBead Borrelia assay were positive in 15 out of 21 patients with pleocytosis in the possible LNB group and, out of these 15 patients, 12 were CXCL13-positive (median 711 pg/mL) (data not shown).

### The overall diagnostic performance of different combinations of tests

An overview of different combinations of tests is presented in Table 6. The highest sensitivity (91%) at an acceptable level of specificity (90%) is obtained by a combination of all three tests with a positive predictive value (PPV) of 84% and a negative predictive value (NPV) of 95% (Table 6). The combination of recomBead Borrelia AI and/or CXCL13 and/or total IgM index also indicate the highest and most appropriate AUC of 0.945 (Table 6).

**Table 6:** Overview of different combinations of tests and their diagnostic performances

	recomBead AI	recomBead AI and/or CXCL13	recomBead AI and/or total IgM^a^	recomBead AI and/or CXCL13 and/or total IgM^a^
Sensitivity (%)	74	88	86	91
Specificity (%)	97	93	95	90
PPV (%)	93	84	91	84
NPV (%)	87	90	93	95
AUC	0.856	0.922	0.923	0.945

AI = antibody index, i.e. intrathecally produced IgG and/or IgM anti-*Borrelia* antibodies detected by the recomBead assay; PPV = positive predictive value; NPV = negative predictive value; AUC = area under the ROC curve

^a^Index calculated from total IgM in serum and CSF

### Clinical aspects of the test results

When analysing the test results in relation to clinical information more in detail, there were a few patients in the non-LNB group (*n* = 7) with elevated recomBead Borrelia AI and/or CXCL13 and/or total IgM index (Table 5). Three of these patients (*n* = 3) were recomBead Borrelia AI-positive but none had elevated IgM index or pleocytosis in CSF. Furthermore, five patients (*n* = 5) had elevated CXCL13 in CSF, with a median value of 330 pg/mL (range 161–424).

One child (*n* = 1) presented with acute idiopathic facial nerve palsy with short duration of symptoms (1–2 days), no pleocytosis but slightly elevated CXCL13 in CSF (161 pg/mL), positive recomBead AI and elevated anti-*Borrelia* antibodies in serum (both recomBead and IDEIA, IgG and IgM). Two more patients (*n* = 2) presented with acute idiopathic facial nerve palsy with short duration of symptoms (1–2 days), no pleocytosis but elevated CXCL13 in CSF (422 and 330 pg/mL respectively), no anti-*Borrelia* antibodies in CSF or serum and no other probable diagnosis. One girl (*n* = 1) had been afflicted with an acute sensorineural hearing loss, had no pleocytosis in CSF, no anti-*Borrelia* antibodies in CSF or serum but had elevated CXCL13 in CSF (424 pg/mL). This patient also remembered a red skin lesion on the neck (possibly undiagnosed EM).

In addition, three patients (*n* = 3) presented with headache (duration 1–2 weeks), no pleocytosis and no anti-*Borrelia* antibodies in CSF. One of these patients had elevated CXCL13 in CSF (237 pg/mL) and two were positive for recomBead Borrelia AI.

Among patients with other diagnosis, a few children (*n* = 3) had elevated CXCL13 and/or total IgM index (Table 5). Two of them presented with fever, headache, meningeal signs, pleocytosis in CSF but no anti-*Borrelia* antibodies in CSF or serum and were diagnosed as clinical viral meningitis (negative PCR for enterovirus). Both patients had elevated total IgM index (0.36 and 0.30, respectively) and one had elevated CXCL13 in CSF (310 pg/mL). One patient had intracranial hypertension, no pleocytosis in CSF, no anti-*Borrelia* antibodies in CSF or serum, no other obvious diagnosis but elevated CXCL13 (204 pg/mL) in CSF.

## Discussion

The results from the present study of well-characterised paediatric LNB patients from a representative clinical setting show that the recomBead Borrelia AI assay has a high specificity of 97% but a lower sensitivity of 74%. However, children with LNB often present with short duration of symptoms (early LNB) and intrathecal anti-*Borrelia* antibody production has not yet started [6, 7]. These children are normally classified as possible LNB, they receive antibiotics and they respond well to treatment [7]. In this present study, we found that the recomBead Borrelia AI was elevated to a higher extent (48%) in the possible LNB group than the IDEIA anti-*Borrelia* antibody assay. Thus, the recomBead Borrelia AI assay is shown here to be superior to the IDEIA assay in children with possible LNB. All recomBead AI-positive patients in the possible LNB group in our study (*n* = 10) also had elevated CXCL13 in CSF and/or total IgM index, further supporting the early LNB diagnosis.

When analysing diagnostic performances of different tests, it is desirable to have a gold standard to confirm the diagnosis and classify patients correctly. However, in laboratory diagnostics of LNB, there is no gold standard due to difficulties in culturing the *Borrelia* spirochete from patient specimens [23]. Consequently, new tests are often compared to current tests that also are a part of the classification of patients, which naturally leads to problems in the interpretation of test results. To compensate for this weakness, it is important to have well-characterised patients, based on relevant and valid clinical information, which we have strived after in this present study. The possible LNB group consisted of patients with neurological symptoms of short duration indicative of LNB, typical season for LNB, no clinical or laboratory signs of viral or bacterial meningitis and no other obvious diagnosis. They all had pleocytosis in CSF with typical mononuclear dominance [21] and they responded well to antibiotic treatment. We, therefore, consider the possible LNB group of children to be representative for early LNB and we believe that the positive recomBead AI results in this group are correctly interpreted as positive compared to the negative results of the IDEIA assay.

In order to further strengthen the sensitivity in early LNB, we wanted to explore both CXCL13 in CSF and total IgM index as early LNB markers and to evaluate whether they could be useful as complementary tools in laboratory diagnostics of LNB in children. Since the chemokine CXCL13 in CSF has proven to be a valid early marker for LNB in previous studies [12, 13], it was suitable for inclusion in analysis of paediatric LNB patients in this present study. Furthermore, the total IgM index has previously shown interesting results in a mixed adult–paediatric LNB patient material [15] and was, therefore, relevant and interesting to include for analysis in our study.

Our results show that both CXCL13 and total IgM index add useful information in combination with the recomBead Borrelia AI assay (Tables 5 and 6). There was also a high concordance between the three different tests among possible LNB patients (Fig. 1). Consequently, to use an antibody test based on several recombinant-specific *Borrelia* antigens (recomBead Borrelia AI) in combination with early markers such as CXCL13 and total IgM index seems to be a promising way to go in laboratory diagnostics of LNB in paediatric patients. Furthermore, since both albumin and IgM in serum and CSF are already included as part of the recomBead Borrelia AI assay, it seems easy and efficacious to use data for the total IgM index as a complementary early marker for LNB in order to further improve sensitivity in LNB diagnostics. Although the results for total IgM index are, to an extent, already included in the recomBead Borrelia AI calculation, it is evident from our results that including total IgM index to the recomBead Borrelia AI results adds diagnostic value.

The serum/CSF samples had been stored in −70 °C from admission (2010–2013) until time for analysis (2015) without being thawed, rendering uncompromised sample quality. Furthermore, samples from all patient groups were handled in the same way and previous studies indicate that serum proteins such as albumin and immunoglobulins, as well as CXCL13, are stable during prolonged storage [15, 24].

Concerning the recomBead Borrelia serum assay, both the sensitivity and specificity were lower than in the recomBead Borrelia AI assay. Among LNB patients with positive recomBead Borrelia AI, only 30 out of 43 (69%) were also positive in serum (IgG and/or IgM), confirming that CSF is required for LNB diagnostics, which is in congruence with previous studies [25]. An invasion from skin to central nervous system without hematogenous phase is another possibility in LNB children with tick bite/EM on the head and neck region and ipsilateral facial nerve palsy [7].

Among LNB patients with pleocytosis in CSF but negative recomBead Borrelia AI, 12 out of 21 were positive in both serum (IgG and/or IgM) and CXCL13. Thus, in some cases, serum antibodies may support the LNB diagnosis. However, one needs to keep in mind that serum antibody results are always ambiguous in interpretation due to uncertainties of cross-reactivity to other infections (elevated IgM), as well as previous LB infections (elevated IgG) [23].

A few non-LNB patients with short duration of symptoms, no pleocytosis and no anti-*Borrelia* antibodies in CSF, but elevated CXCL13 in CSF, were reported in detail since they are interesting and may represent patients with very early LNB being misdiagnosed as non-LNB. Since the role of CXCL13 is to recruit inflammatory cells into the central nervous system [12], it would be rational to believe that elevated CXCL13 could be detectable before pleocytosis in CSF in a few patients with very early LNB, but this needs further investigation in future studies.

## Conclusion

The recomBead Borrelia antibody index (AI) assay performs with moderate sensitivity in paediatric Lyme neuroborreliosis (LNB) patients and the major advantage seems to be increased sensitivity in the possible LNB group compared to the IDEIA assay. Thus, the two assays complement each other in sensitivity but have comparable high specificity. The recomBead Borrelia AI assay is more expensive and requires measurements of albumin and total immunoglobulin levels in serum and cerebrospinal fluid (CSF). Therefore, it may be used in IDEIA-negative paediatric patients with CSF pleocytosis (potential early LNB cases). The diagnostic sensitivity may be further increased by using a combination of early markers, such as CXCL13 in CSF and total IgM index.
